# Identification of the orphan gene Prod 1 in basal and other salamander families

**DOI:** 10.1186/s13227-015-0006-6

**Published:** 2015-04-11

**Authors:** Jie Geng, Phillip B Gates, Anoop Kumar, Stefan Guenther, Acely Garza-Garcia, Carsten Kuenne, Peng Zhang, Mario Looso, Jeremy P Brockes

**Affiliations:** State Key Laboratory of Biocontrol, College of Ecology and Evolution, School of Life Sciences, Sun Yat-Sen University, Guangzhou, 510006 China; Institute of Structural and Molecular Biology, Division of Biosciences, UCL, Gower Street, London, WC1E 6BT UK; Max-Planck-Institute for Heart and Lung Research, Ludwigstrasse 43, 61231 Bad Nauheim, Germany; National Institute for Medical Research, The Ridgeway, Mill Hill, London, NW7 1AA UK

**Keywords:** Three-finger protein, Phylogeny, Limb regeneration, Plethodontid, Hynobiid

## Abstract

**Background:**

The urodele amphibians (salamanders) are the only adult tetrapods able to regenerate the limb. It is unclear if this is an ancestral property that is retained in salamanders but lost in other tetrapods or if it evolved in salamanders. The three-finger protein Prod 1 is implicated in the mechanism of newt limb regeneration, and no orthologs have been found in other vertebrates, thus providing evidence for the second viewpoint. It has also been suggested that this protein could play a role in salamander-specific aspects of limb development. There are ten families of extant salamanders, and Prod 1 has only been identified in two of them to date. It is important to determine if it is present in other families and, particularly, the basal group of two families which diverged approximately 200 MYA.

**Findings:**

We have used polymerase chain reaction (PCR) to identify Prod 1 in a Chinese hynobiid species *Batrachuperus longdongensis.* We obtained an intestinal transcriptome of the plethodontid *Aneides lugubris* and, from this, identified a primer which allowed PCR of two Prod 1 genes from this species. All known Prod 1 sequences from nine species in four families have been aligned, and a phylogenetic tree has been derived.

**Conclusions:**

Prod 1 is found in basal salamanders of the family Hynobiidae, and in at least three other families, so it may be present in all extant salamanders. It remains a plausible candidate to have been involved in the origins of limb regeneration, as well as the apomorphic aspects of limb development.

## Findings

### Introduction

Prod 1 was originally identified as a retinoid-inducible gene expressed during newt limb regeneration [1]. It is a member of the three-finger protein superfamily that is attached to the cell surface with a glycosylphosphatidylinositol (GPI) anchor and is expressed in the adult newt limb in a shallow proximodistal gradient [2]. It has been shown to have activities during regeneration that are relevant for both nerve dependence and positional identity of the limb blastema [3,4]. The 3D structure of the protein in solution has been solved by NMR and has a distinctive uninterrupted 12-residue α-helical stretch in the third finger [5]. The molecular phylogeny, based on both sequence and structural criteria, indicates that Prod 1 has no known orthologues in other vertebrate taxa. In particular, exhaustive searches and phylogenetic analyses of three-finger proteins (TFPs) from *Xenopus* and zebrafish suggest that no Prod 1 ortholog is present [6]. Thus, it is apparently a salamander orphan gene implicated in limb regeneration.

Salamanders (urodeles) are the only adult tetrapods able to regenerate the limb. It is unclear if limb regeneration evolved in salamanders or if it is an ancestral property for vertebrates that is retained in salamanders and lost in other tetrapods [6]. The example of Prod 1, as well as other less studied candidates derived from proteomic or transcriptomic analysis of salamander regeneration [7,8], provides evidence for the hypothesis of local evolution, although many questions remain to be answered [9]. It has also been suggested that Prod 1 could be implicated in salamander-specific aspects of limb development such as pre-axial dominance [10], which is considered to be apomorphic for urodeles [11].

There are ten families of extant salamanders, and a recent phylogenetic analysis, based on 30 different nuclear genes in 19 species, has concluded that the basal group of salamanders are the Cryptobranchoidea encompassing the two families Hynobiidae and Cryptobranchidae [12]. Limb regeneration has been detected in these salamanders [13], and the question has been raised as to whether Prod 1 is present in this group and, hence, presumably in the other families [14]. This protein has only been studied to date in newt and *Ambystoma* species (families Salamandridae and Ambystomatidae) [15], and we report here that it is also present in the Hynobiidae and in one other family, the Plethodontidae, the most derived and most speciose family of salamanders. During the preparation of this manuscript, transcriptomic data from *Hynobius chinensis* became available [16], and we have also included the sequence of Prod 1 from this species in our alignment and analyses. These results support the hypothesis that Prod 1 is present in all extant salamanders and is implicated in the evolution of limb regeneration.

### Results and discussion

**Figure 1:**
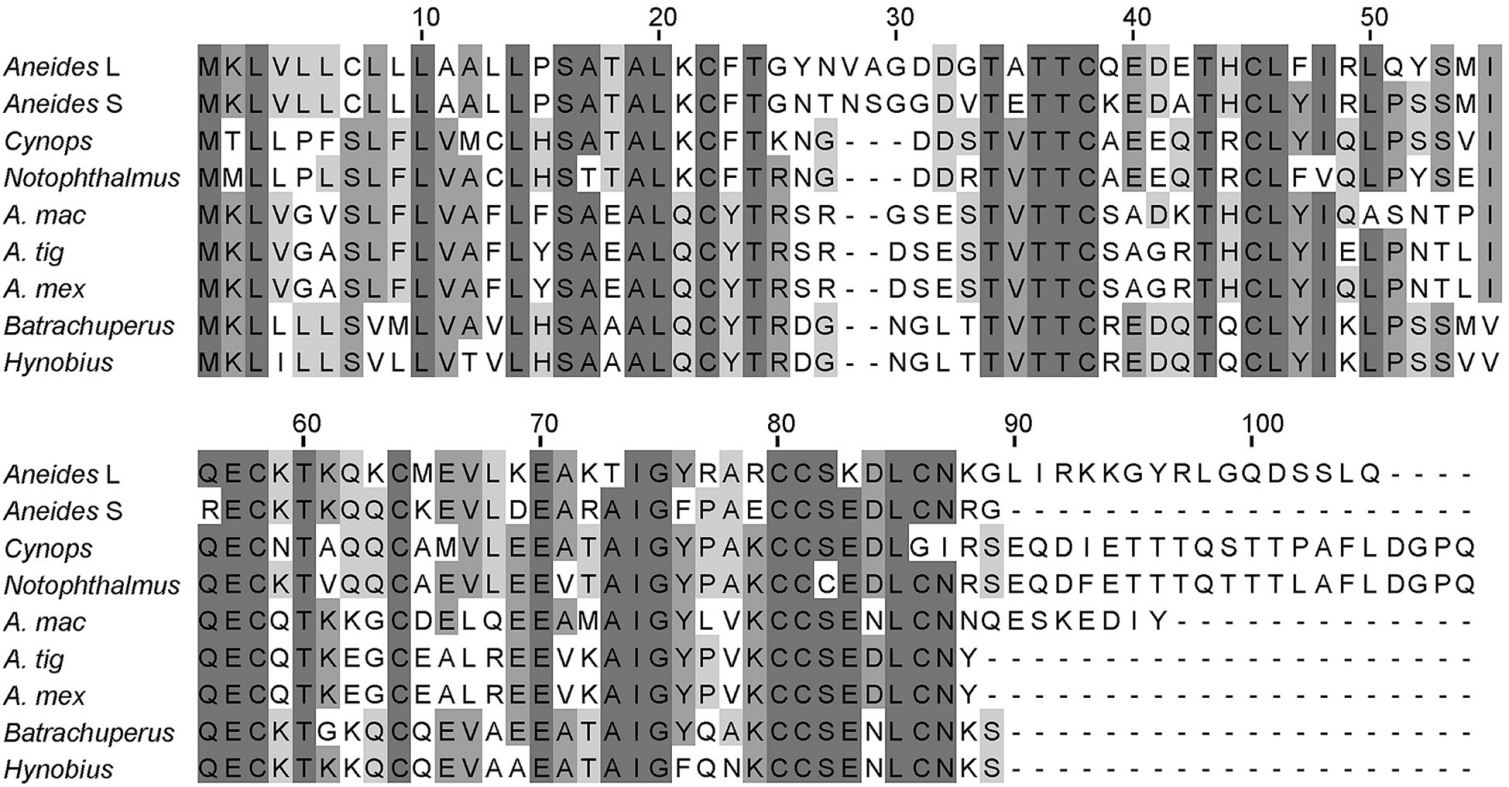
Alignment of nine Prod 1 sequences in four families of salamanders (Salamandridae, Ambystomatidae, Plethodontidae, Hynobiidae). In order to obtain *A. lugubris* Prod 1, the oligonucleotide TGCTGCCATGCCCAAAACAGGAAGCCATGA (obtained from the intestinal transcriptome) was extended by RACE cDNA amplification with intestinal cDNA and gave the two related sequences shown here. For *B. longdongensis*, two cycles of nested PCR were performed on intestine cDNA, the first with forward primer TCARCYACAGCNYTRMAATG and the 5′ RACE primer ARCAGCAYTTKRCTGGATAKCCAATGG, and the second with forward primer CGASRKCACTGNRACYACMTG and reverse primer GTTTKRCATTCYYGWATCDBAG. For *C. orientalis*, the degenerate 5′ RACE primer AGATCCTCSGARCAGCAYTTTRCTGGATA and the 3′ RACE primer CTGGTGATGTGCCTACACTCAGCTACAGCT were used on limb cDNA in order to obtain the full length Prod 1 sequence. The detailed procedures for cloning are available on request. The GenBank accession numbers are KP686220 (*C. orientalis*), KP686221 (*B. longdongensis*), KP686222 (Aneides L), KP686223 (Aneides S). The *A. lugubris* transcriptome is available on open access at https://bioinformatics.mpi-bn.mpg.de/library.

We synthesised cDNA from the total RNA extracted from the intestine of the Chinese hynobiid, *Batrachuperus longdongensis*, the Longdong stream salamander. A nested polymerase chain reaction (PCR) strategy based on conserved primers was used to amplify the full-length sequence for Prod 1 (see legend to Figure 1). Intestinal cDNA was also synthesised from the plethodontid *Aneides lugubris*, the arboreal salamander, but repeated attempts to use nested PCR to obtain Prod 1 were not successful. Therefore, the intestinal transcriptome was obtained, and a single short-sequence read yielded an oligonucleotide primer potentially related to Prod 1. This was extended in both orientations from the cDNA of a single individual to give two related Prod 1 sequences referred to as short and long (Figure 1). These differ in the presence of a C terminal extension of 15 residues and also at several internal positions. The expression of long and short forms was analysed in various tissues of *A. lugubris* by PCR and is shown relative to the limb in Table 1. Although they were both expressed in the limb, they were regulated quite markedly in other tissues such as liver and heart (Table 1). It seems likely from the sequence and expression data that the long and short forms are different genes subject to independent regulation, although we cannot exclude that they are derived by alternative splicing. The functional significance of the two forms is unknown.

**Table 1:** **Expression of long and short forms of Prod 1 in tissues of** ***A*** **.** ***lugubris***

**Tissue**	**Long**	**Short**
Limb	4.50	1
Tail	4.77	0.75
Liver	1,456	12.80
Heart	0.23	1.86
Brain	4.37	2.97
Spinal cord	10.44	4.66
Intestine	16.69	13.34

Real-time PCR was performed in triplicate on two independent cDNA samples for each tissue. The primers for the short form were GGTTATAACGTTGCTGGTGAC and GTACATGTTGATGCTGCCAT; the primers for the long form were GGTAATACGAATTCTGGTGGT and GTACATGTTGATGCTGCCAT. The long and short forms were cloned in tandem into a single plasmid, which was used to calibrate a standard curve for the PCR analysis. The results were normalised with respect to the expression of GAPDH and expressed with the level of the short form in the limb as unity. Note that relative expression varies markedly in different tissues.

**Figure 2:**
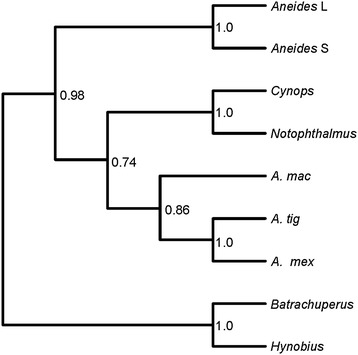
Phylogenetic tree for the Prod 1 sequences in Figure 1. Trees were computed by maximum likelihood and Bayesian inference, with equivalent results. Maximum likelihood trees were built using PhyML [21] and jModeltest [22] for model selection; support for clades was evaluated with 1,000 bootstrap pseudoreplicates. Bayesian analyses were carried out with MrBayes v.3.1.2 [23], for a total of 50,000 generations, sampling every 50 generations. Substitution models were estimated during the analysis by model-jumping with a proportion of invariable sites.

The currently available Prod 1 sequences are aligned in Figure 1, which also includes the previously unpublished sequence for the newt species *Cynops orientalis*, and a phylogenetic tree derived by Bayesian analysis is shown in Figure 2. The addition of this new, more divergent, set of Prod 1 sequences to our public database searches and phylogenetic analyses has not revealed any TFP superfamily member that might correspond to a non-salamander Prod 1. In view of the conserved N terminal signal sequence, Prod 1 presumably enters the secretory pathway in all salamanders but only in newts does it acquire a GPI anchor via the C terminal anchor signal sequence (residues 90 to 109). The significance of this difference for the mechanism of limb regeneration is unclear. The extension in the long plethodontid form is not predicted to be an anchor signal, and thus, Prod 1 in all species apart from newts would be expected to be secreted. The long form is a result of a one base insertion that changes the reading frame of the protein and bypasses the stop codon, so it is not surprising that the resulting extension is not an anchor signal.

It is noteworthy that Prod 1 is found in two species of basal salamander and was therefore presumably present in the last common ancestor of crown group salamanders at the time of divergence, estimated to be at the beginning of the Jurassic. In recent analysis of fossils, evidence for the salamander-specific phenotypes of pre-axial dominance [17,11], and limb regeneration [18], has been detected in dissorophoid temnospondyl amphibians of the early Permian (300 to 290 MYA). This may have been close to the origin of salamanders in tetrapod evolution [19], and it is possible that this also coincided with the origin of Prod 1 [20]. Prod 1 could have been present in Lower Permian dissorophoids and subsequently lost in anurans.

## Abbreviations

3D: three-dimensional
A. mac: *Ambystoma maculatum*
A. mex: *Ambystoma mexicanum*
A. tig: *Ambystoma tigrinum*
GAPDH: glyceraldehyde phosphate dehydrogenase
GPI: Glycosylphosphatidylinositol
MYA: million years ago
PCR: polymerase chain reaction
TFP: three-finger protein

## References

[1] Morais Da Silva S, Gates PB, Brockes JP (2002). The newt ortholog of CD59 is implicated in proximodistal identity during amphibian limb regeneration. Dev Cell.

[2] Kumar A, Gates PB, Brockes JP (2007). Positional identity of adult stem cells in salamander limb regeneration. C R Biol.

[3] Echeverri K, Tanaka EM (2005). Proximodistal patterning during limb regeneration. Dev Biol.

[4] Kumar A, Godwin JW, Gates PB, Garza-Garcia AA, Brockes JP (2007). Molecular basis for the nerve dependence of limb regeneration in an adult vertebrate. Science.

[5] Garza-Garcia A, Harris R, Esposito D, Gates PB, Driscoll PC (2009). Solution structure and phylogenetics of Prod1, a member of the three-finger protein superfamily implicated in salamander limb regeneration. PLoS One.

[6] Garza-Garcia AA, Driscoll PC, Brockes JP (2010). Evidence for the local evolution of mechanisms underlying limb regeneration in salamanders. Integr Comp Biol.

[7] Looso M, Michel CS, Konzer A, Bruckskotten M, Borchardt T, Kruger M (2012). Spiked-in pulsed in vivo labeling identifies a new member of the CCN family in regenerating newt hearts. J Proteome Res.

[8] Looso M, Preussner J, Sousounis K, Bruckskotten M, Michel CS, Lignelli E (2013). A de novo assembly of the newt transcriptome combined with proteomic validation identifies new protein families expressed during tissue regeneration. Genome Biol.

[9] Mihaylova Y, Aboobaker AA (2013). What is it about ‘eye of newt’?. Genome Biol.

[10] Brockes JP, Gates PB (2014). Mechanisms underlying vertebrate limb regeneration: lessons from the salamander. Biochem Soc Trans.

[11] Frobisch NB, Shubin NH (2011). Salamander limb development: integrating genes, morphology, and fossils. Developmental dynamics : an official publication of the American Association of Anatomists.

[12] Shen XX, Liang D, Feng YJ, Chen MY, Zhang P (2013). A versatile and highly efficient toolkit including 102 nuclear markers for vertebrate phylogenomics, tested by resolving the higher level relationships of the caudata. Mol Biol Evol.

[13] Griffin PC, Solkin VA (1995). Ecology and conservation of Onychodactylus fischeri (Caudata, Hynobiidae) in the Russian Far East. Asiatic Herpetol Res.

[14] Sanchez AA (2012). Q&A: what is regeneration, and why look to planarians for answers?. BMC Biol.

[15] Blassberg RA, Garza-Garcia A, Janmohamed A, Gates PB, Brockes JP (2011). Functional convergence of signalling by GPI-anchored and anchorless forms of a salamander protein implicated in limb regeneration. J Cell Sci.

[16] Che R, Sun Y, Wang R, Xu T (2014). Transcriptomic analysis of endangered Chinese salamander: identification of immune, sex and reproduction-related genes and genetic markers. PLoS One.

[17] Frobisch NB, Carroll RL, Schoch RR (2007). Limb ossification in the Paleozoic branchiosaurid Apateon (Temnospondyli) and the early evolution of preaxial dominance in tetrapod limb development. Evol Dev.

[18] Frobisch NB, Bickelmann C, Witzmann F (2014). Early evolution of limb regeneration in tetrapods: evidence from a 300-million-year-old amphibian. Proceedings Biological sciences/The Royal Society.

[19] Schoch RR. Amphibian evolution: the life of early land vertebrates. Wiley Blackwell: Topics in Paleobiology; 2014

[20] Brockes JP, Kumar A, Simon A (2015). Variation in salamanders: an essay on genomes, development and evolution. Salamanders in Regeneration Research: Methods and Protocols.

[21] Guindon S, Dufayard JF, Lefort V, Anisimova M, Hordijk W, Gascuel O (2010). New algorithms and methods to estimate maximum-likelihood phylogenies: assessing the performance of PhyML 3.0. Syst Biol.

[22] Darriba D, Taboada GL, Doallo R, Posada D (2012). jModelTest 2: more models, new heuristics and parallel computing. Nat Methods.

[23] Ronquist F, Huelsenbeck JP (2003). MrBayes 3: Bayesian phylogenetic inference under mixed models. Bioinformatics.

